# Tracking Eye Movements During Sleep in Mice

**DOI:** 10.3389/fnins.2021.616760

**Published:** 2021-02-25

**Authors:** Qingshuo Meng, Xinrong Tan, Chengyong Jiang, Yanyu Xiong, Biao Yan, Jiayi Zhang

**Affiliations:** Institutes of Brain Science, State Key Laboratory of Medical Neurobiology, MOE Frontiers Center for Brain Science, Department of Ophthalmology, Zhongshan Hospital, Fudan University, Shanghai, China

**Keywords:** eye movement tracking, sleep–wake cycle, tonic REM, phasic REM, biocompatibility

## Abstract

Eye movement is not only for adjusting the visual field and maintaining the stability of visual information on the retina, but also provides an external manifestation of the cognitive status of the brain. Recent studies showed similarity in eye movement patterns between wakefulness and rapid eye movement (REM) sleep, indicating that the brain status of REM sleep likely resembles that of awake status. REM sleep in humans could be divided into phasic REM and tonic REM sleep according to the difference in eye movement frequencies. Mice are the most commonly used animal model for studying neuronal and molecular mechanisms underlying sleep. However, there was a lack of details for eye movement patterns during REM sleep, hence it remains unknown whether REM sleep can be further divided into different stages in mice. Here we developed a device combining electroencephalogram (EEG), electromyogram (EMG) as well as eye movements recording in mice to study the eye movement patterns during sleep. We implanted a magnet beneath the conjunctiva of eye and tracked eye movements using a magnetic sensor. The magnetic signals showed strong correlation with video-oculography in head-fixed mice, indicating that the magnetic signals reflect the direction and magnitude of eye movement. We also found that the magnet implanted beneath the conjunctiva exhibited good biocompatibility. Finally, we examined eye movement in sleep–wake cycle, and discriminated tonic REM and phasic REM according to the frequency of eye movements, finding that compared to tonic REM, phasic REM exhibited higher oscillation power at 0.50 Hz, and lower oscillation power at 1.50–7.25 Hz and 9.50–12.00 Hz. Our device allowed to simultaneously record EEG, EMG, and eye movements during sleep and wakefulness, providing a convenient and high temporal-spatial resolution tool for studying eye movements in sleep and other researches in mice.

## Introduction

Eye movements help human to align fovea to the target with attention and stabilize the vision on retina and is precisely regulated by visual, somatosensory, vestibular, and other systems (Sweeney et al., 2007; Kheradmand et al., 2016; Shemesh and Zee, 2019; Billington et al., 2020). Meanwhile, eye movements are also considered as an external manifestation of cognitive activity in the brain, such as learning and memory (Duhamel et al., 1992), attention (Kustov and Robinson, 1996), and decision making (Raposo et al., 2012), and could be utilized as an indicator for functional evaluation of nervous system. Therefore, the measurements and analysis of eye movements can provide insights for understanding cognitive functions as well as pathogenesis and therapeutic mechanisms of psychological diseases.

Sleep is a dynamic process with complex neural activity and plays an important role in metabolism, mood and memory (Pace-Schott et al., 2015; Petit et al., 2015; O’Leary et al., 2017; Acosta, 2019). There are two basic states of sleep characterized by eye movements, namely rapid eye movement (REM) and non-REM sleep. In Rodents, REM sleep was identified as theta band (6–10 Hz) dominant EEG, elevated theta/delta (0.65–4 Hz) power ratio, and high eye movement frequency. Non-REM sleep was identified as delta band (0.65–4 Hz) dominant EEG, low theta/delta power ratio, and low eye movement frequency (Jouvet, 1965; Mann and Roschke, 1997; Weber and Dan, 2016). While in human, non-REM sleep could further be divided into deeper and lighter sleep states, and REM sleep also had different microstates. Recent studies in rodents and human suggested that the eyes were inner indicator of brain state during sleep (Andrillon et al., 2015). In non-REM sleep, pupil size dynamics is couple with sleep depth in mice (Yuzgec et al., 2018). In REM sleep, there are two microstates called phasic REM and tonic REM. The phasic REM sleep is characterized by bursts of eye movements, while tonic REM sleep had less eye movements. These two microstates had different arousal thresholds and spontaneous oscillation activity. Phasic REM sleep showed higher threshold for awakening than tonic REM sleep (Brankačk et al., 2012). In spontaneous oscillation activity, tonic REM sleep showed elevated high alpha and beta band, while phasic REM sleep showed the predominance of slow delta, theta, and higher gamma band (Ermis et al., 2010; Simor et al., 2020). Mice are the most common animal model for studying the mechanism of sleep and sleep associated cortical dynamics (Liu et al., 2016; McKillop et al., 2018; Kam et al., 2019), but there is still lack of methods to measure eye movements in mice during sleep. Developing a device to track eye movements with eyelid closed would be useful to unveil the role of eye movements in sleep depth and cortical processing during sleep.

The average diameter of eyeball in mice was only 3.4 mm (Remtulla and Hallett, 1985), which was much smaller than other mammalian animal models, such as cat, rabbit, and monkey (Carrington and Woodward, 1986; Bozkir et al., 1997; Ross and Kirk, 2007), making it difficult to track eye movements precisely. One of the non-invasive methods for eye movement detection is electrooculography (EOG) measuring electric potential differences with pairs of electrodes pasted either below and above the sclera of the eye (Lin et al., 2015; Creel, 2019; Jia and Tyler, 2019). However, EOG do not measure eye movements directly and was susceptible to inaccuracies (Arden and Kelsey, 1962). Video-oculography is also non-invasive method for tracking eye movements in head-fixed animals and requires eye open during recording, hence unsuitable to track eye movements during sleep (Mitchiner et al., 1976; Stahl et al., 2000; Kimmel et al., 2012; Wallace et al., 2013; Brooks et al., 2019). Eye coil is the gold standard for tracking eye movements by measuring electric currents which generated through electromagnetic induction. The eye coil of wire is surgically implanted under the conjunctiva around the eye and the animal is placed in an alternating, high-frequency magnetic field, but it is difficult to be implanted into small eyes (Robinson, 1963; Fuchs and Robinson, 1966; Judge et al., 1980; Koekkoek et al., 1997; Boyden and Raymond, 2003). Compared with video-oculography, Payne and Raymond (2017) developed a magnetic eye tracking system with lightweight and high spatial and temporal resolution magnetic sensor that measures eye position of <0.1°, providing a powerful tool to record eye movements during sleep in mice.

In this study, we developed a device combining electroencephalogram (EEG) electrodes, electromyogram (EMG) electrodes, and magnetic sensor. We found that the signal of eye movements recorded by the device showed strong correlation with video-oculography in head-fixed mice, and the magnet implanted showed good biocompatibility. Finally, we utilized the device to record eye movements during sleep and examined eye movements frequency in REM sleep, non-REM sleep, and wakefulness. Our device simultaneously records EEG, EMG, and eye movements during sleep and wakefulness, providing a powerful tool for further researches in different sleep stages.

## Results

### Integrated Device for EEG, EMG, and Eye Movements Recording

**FIGURE 1:**
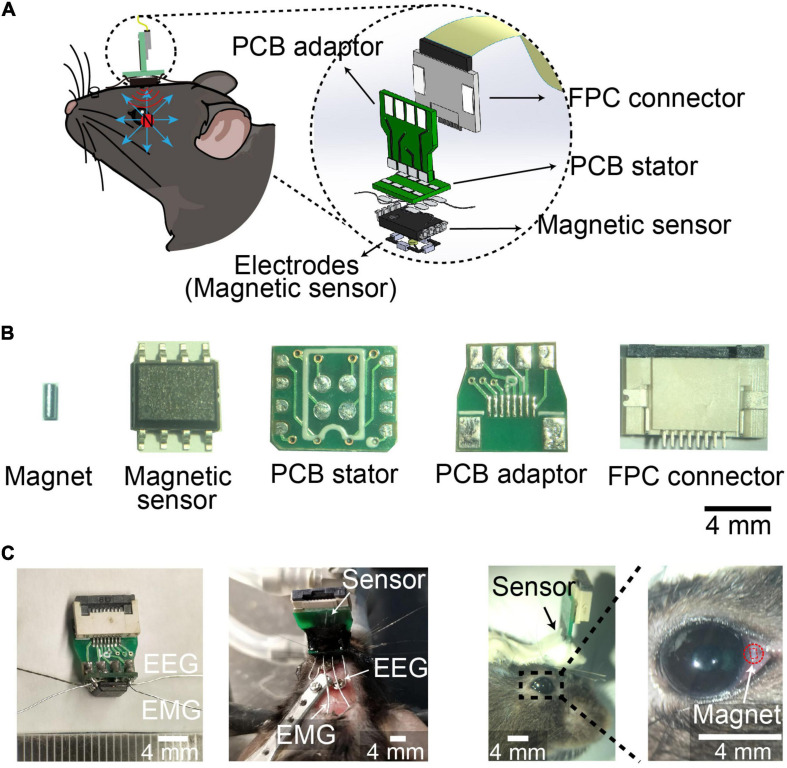
Device for EEG, EMG, and eye movements recording during sleep. **(A)** Schematic of implanted device recording EEG, EMG and eye movements. **(Left)** device mounted on the head of mice with magnet implanted into conjunctiva. **(Right)** 3D Model showing the components of the device. **(B)** Photograph of magnet, magnetic sensor, PCB stator, PCB adaptor, and FPC connector. Scale bar, 4 mm. **(C)** Photograph of the device **(Left)**, head-mounted device with electrodes implanted in mice **(middle)**, and magnet implanted into conjunctiva **(Right)**. Red square indicated the outline of magnet implant. Scale bar, 4 mm.

The spatial resolution of magnetic eye tracking was 0.098°, while the spatial resolution by eye coil and video-oculography was 0.091° and 0.23°, respectively, suggesting the spatial resolution of magnetic eye tracking was as high as eye coil method, and better than video-oculography (Iwashita et al., 2001; Rodriguez et al., 2001; Kaneko et al., 2010; Schwarz et al., 2013; Liu et al., 2018). In order to record eye movements during different stages in sleep–wake cycle, we designed an integrated device combining EEG and EMG electrodes with neodymium (NdFeB) magnetic sensor-based eye movements detector. The magnetic field detected by the sensor changes when the eyeball moved (Figure 1A). We soldered EEG electrodes, EMG electrodes and magnetic sensor onto a customer-made printed circuit board (PCB) stator, which was connected to a flexible printed circuit (FPC) connector through a PCB adaptor (Figure 1B). The integrated device was mounted onto the skull with dental cement, with EEG electrodes implanted onto the skull and EMG electrodes implanted into the neck muscle, and NdFeB magnet was implanted into conjunctiva in mice (Figure 1C). We further examined the physiological range of the signal in vitro according to the distance between magnet and magnetic sensor in vivo. We found that the signals could be detected no matter the magnet rotated in any angle. And when increasing the horizontal distance between magnet and magnetic sensor, the signal was also decreased, but still be detected (Supplementary Figure 1). These data indicated that the variation of the signal recorded by magnet sensor might reflect eye movement *in vivo*.

### Eye Movements Tracking by Magnetic Sensor

**FIGURE 2:**
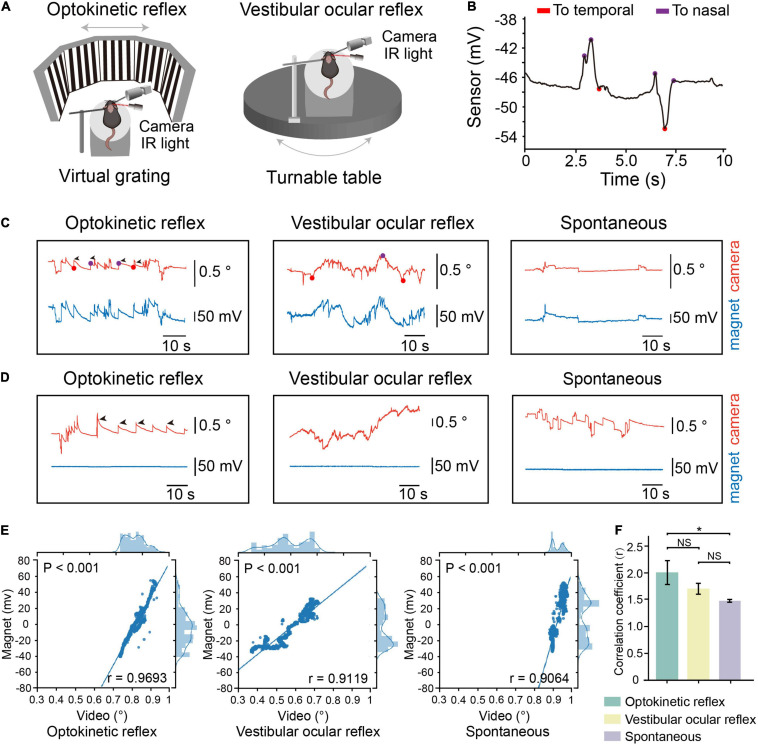
Comparation of eye movements tracking by the device and video-oculography. **(A)** Schematic representation of experimental setup for tracking eye movements during optokinetic reflex (OKR) recording **(Left)** or vestibular ocular reflex (VOR) recording **(Right)**. **(B)** Motion direction of eye analyzed from eye movements raw trace. Red dots, the timepoint when eye move to temporal. Purple dots, the timepoint when eye move to nasal. **(C)** Example of OKR induced eye movements **(left)**, VOR induced eye movements **(middle)** or spontaneous eye movements from one mouse with magnet implanted on conjunctiva. Red line: relative eye position measured by video-oculography. Blue line: relative eye position measured by magnetic sensor. **(D)** Example of OKR induced eye movements **(left)**, VOR induced eye movements **(middle)** or spontaneous eye movements from one mouse with magnet implanted on skull. **(E)** Scatterplot of eye position recorded by magnetic sensor and video-oculography for one representative mouse. Solid line represents fitted linear regression. The curve in top axis represents eye position distribution recorded by video-oculography. The curve in right axis represents eye position distribution recorded by magnetic sensor. **(F)** Mean correlation coefficients between signals by video-oculography and magnetic approaches during different eye movement patterns (*n* = 4, data were first processed with Fisher z transformation and one-way repeated measures ANOVA followed by post hoc comparison with Tukey’s correction was performed afterward, NS: not significant). Data are expressed as mean ± SEM, *p < 0.05.

We first conducted simultaneous magnetic eye tracking and video-oculography while inducing optokinetic, vestibular-ocular eye movements to examine the precision of eye movements detection (Figure 2A). When the eyes of mice moved toward the temporal or nasal sides, we can discriminate the directions of eye movements according to the recorded signals (Figure 2B). We found that the magnetic signals synchronized with the tracked pupillary positions in video-oculography (Figure 2C). Meanwhile, we mounted the magnet onto the skull so that there is no relative movement between the magnet and the sensor (control group). We did not see any change in the magnetic signals in the control group (Figure 2D). We also found strong correlation between the magnetic signals and tracked pupillary positions in all the recorded mice (Figures 2E,F and Supplementary Figure 2). These data suggested that the magnetic sensing system is capable of eye movements detection. Meanwhile, the mean values of correlation coefficients between magnetic signals and pupillary positions in video-oculography was 0.9624 in optokinetic reflex (OKR), 0.9482 in vestibular ocular reflex (VOR) and 0.9030 in spontaneous eye movements, and the mean values of correlation coefficients in OKR was significantly higher than in spontaneous eye movements (Figure 2F), and the data points appears to be comprised of two clusters in spontaneous eye movements. These differences might be due to the complex and variable patterns in the spontaneous eye movements in comparison to the stereotyped eye movements characteristic of OKR and VOR. Because OKR and VOR are artificially induced regular eye movement, that is, the eyeball slowly moves from nasal or temporal side to the opposite side, and then returns. However, there is no regular eye movement in spontaneous eye movement mode, and the speed, direction and position are uncertain.

### Biocompatibility of Magnet Implant

**FIGURE 3:**
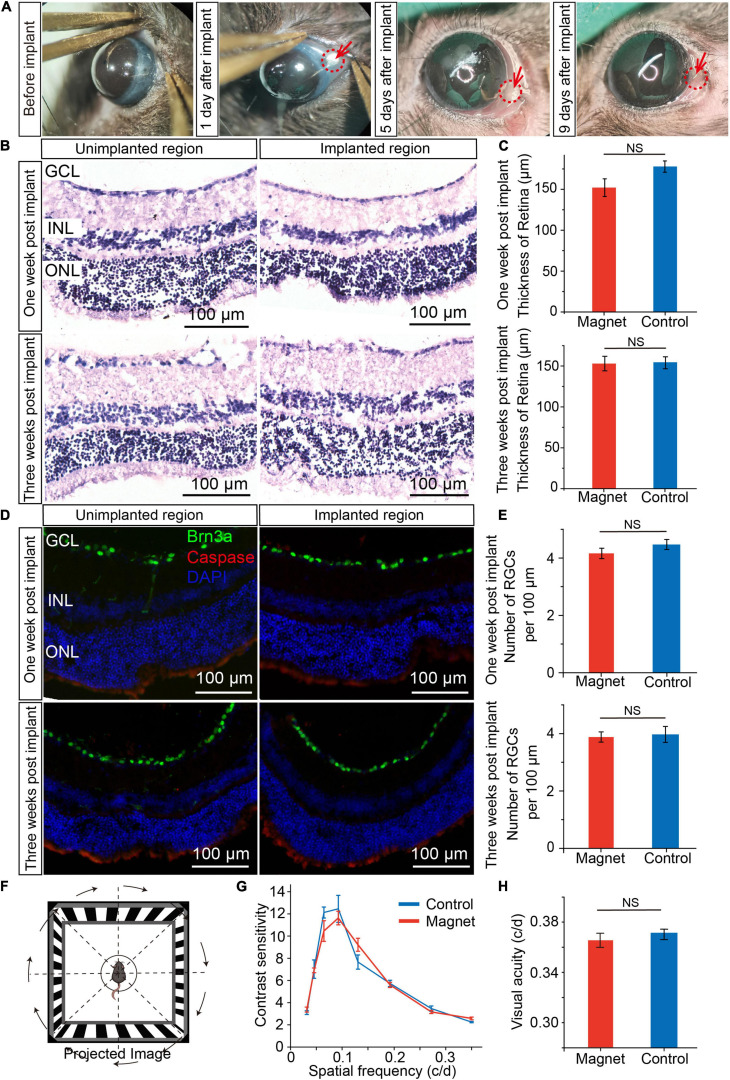
Biocompatibility of magnet implanted in conjunctiva. **(A)** The corneal status before and after magnet implant in one representative mouse. **(B)** Representive HE staining of retina sections one/three weeks post implant after magnet implanted. **(Left)** the region with no magnet implanted; **(Right)** the region with magnet implanted. GCL, ganglion cell layer; INL, inner nuclear layer; ONL, outer nuclear layer. Scale bars, 100 μm. **(C)** Comparation of retinal thickness between magnet implanted retina and no magnet implanted retina (*n* = 5). **(D)** Representive immunofluorescence staining of retina one/three weeks post implant after magnet implanted. Green: Brn3a; red: caspase; blue: DAPI. Scale bars, 100 μm). **(E)** Comparation of number of retina ganglion cells (RGC) per 100 μm in GCL between magnet implanted retina and no magnet implanted retina (*n* = 5). **(F)** Schematic for optomotor response. **(G)** Contrast sensitivity between control mice (*n* = 5) and mice with magnet implant (*n* = 5). **(H)** Visual acuity between control mice (*n* = 5) and mice with magnet implant (*n* = 5, student’s *t*-test, NS, not significant). Data are expressed as mean ± SEM.

To check whether there was corneal injury after magnet implant within the conjunctiva, we examined the corneal status daily, and found that there were signs of redness three days after implant, but seven days after implant, the redness disappeared (Figure 3A and Supplementary Figure 3). And the magnet implant was in place 3 months after implant (Supplementary Figure 4). To examine whether magnet implant in the conjunctiva cause any damage in the retina, we compared the unimplanted and implanted regions in retinal sections by hematoxylin-eosin (H&E) staining (Figure 3B). One and three weeks after magnet implant surgery, no inflammation or change in retinal thickness was detected in the implanted region (Figure 3C). We also found no caspase-expressing cells in the implanted region (Figure 3D). By analyzing number of retinal ganglion cells (RGC) per 100 μm in ganglion cell layer (GCL) with transverse section, we did not see any decrease in RGCs numbers one or three weeks after implant (Figure 3E). We then assessed the visual function and found there were no differences in visual acuity and contrast sensitivity between control mice and mice with magnet implant (Figures 3F–H). These data suggested that the NdFeB magnet had good biocompatibility.

### Eye Movements Detection During Sleep–Wake Cycle

**FIGURE 4:**
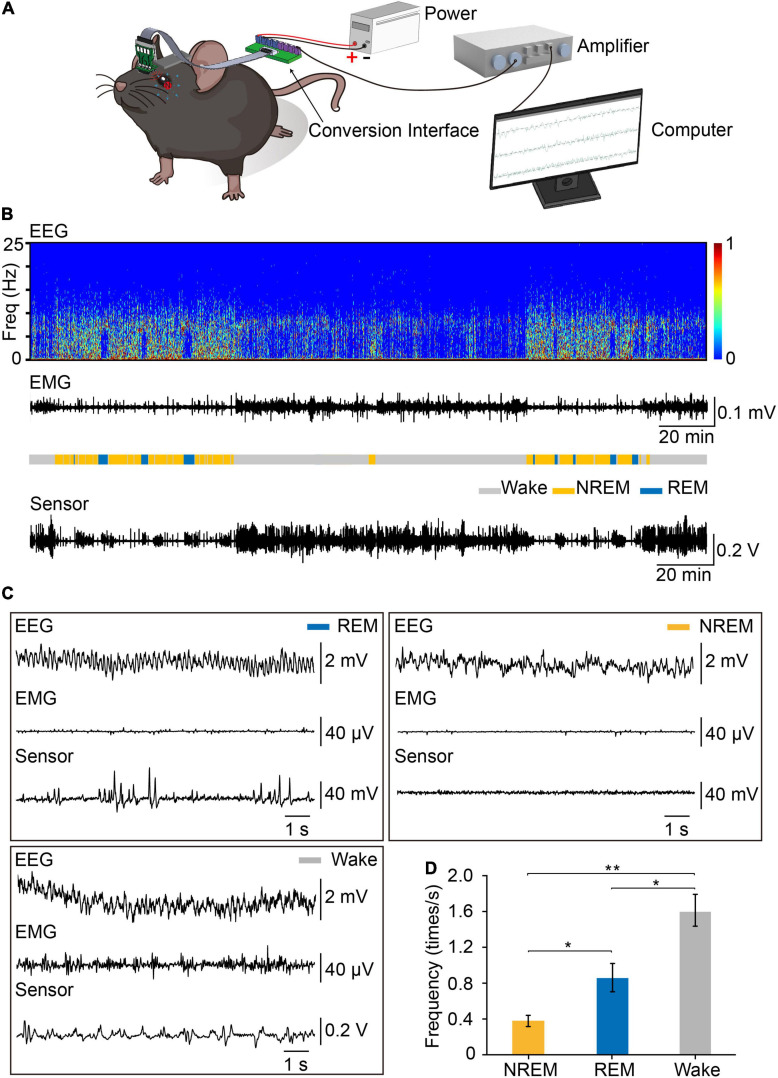
Recording EEG, EMG and eye movements by the device during the sleep–wake cycle. **(A)** Set-up of system for recording EEG, EMG, and eye movements. The signals of EEG, EMG, and eye movements were detected by the device, transferred through FPC, amplified through an amplifier and recorded by Spike2 for analysis. **(B)** Representative data showed EEG power spectrogram, electromyogram amplitude, brain states, and eye movements curve. Sleep pattern of representative data: Wake:147 min, NREM: 70 min, REM: 23 min. **(C)** Example of EEG, EMG, and eye movements raw traces during REM **(left)**, NREM **(right)**, and wakefulness **(left)**. **(D)** Eye movements frequency during different brain states (*n* = 3, One-way repeated measures ANOVA followed by *post hoc* comparison with Tukey’s correction). Data are expressed as mean ± SEM, *p < 0.05, **p < 0.01.

To record eye movements during sleep–wake cycle, we implanted the integrated device into mice, tracking the eye movements using the magnetic sensor and determining the brain states using simultaneous EEG and EMG signals (Figures 4A,B). The spontaneous eye movement was recorded when the mice were awake and freely moving. It appeared that the amplitude and frequency of spontaneous eye movement were significantly larger than other sleep stages (Figure 4B). We found that there was no difference in the daily amounts of wake, REM and NREM between the control mice and the mice with magnet implant (Supplementary Figure 5). As previously reported, the frequency of eye movements during REM sleep was much higher than that during NREM sleep (Hirshkowitz, 2004). Previous studies indicated the frequency of eye movements is higher during REM sleep than wakefulness in human (Andrillon et al., 2015). However, in mice, we found that the frequency of eye movements during wakefulness was higher than that in REM sleep (Figures 4C,D).

### Identification of Tonic and Phasic REM Sleep

**FIGURE 5:**
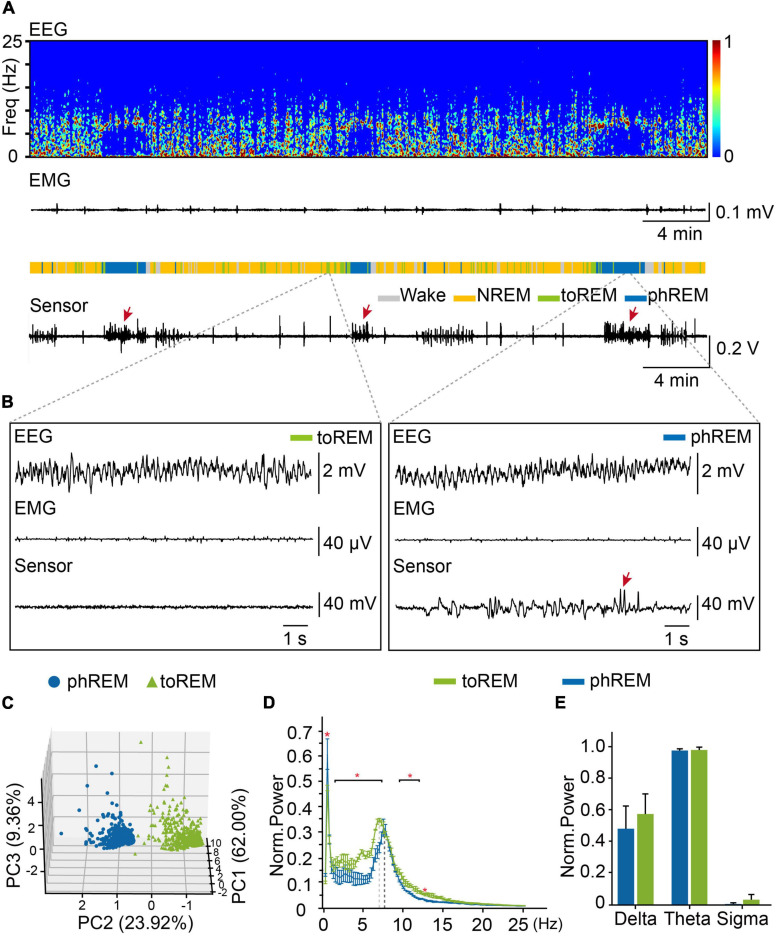
Tonic REM and phasic REM sleep in mice. **(A)** Representative data showed EEG power spectrogram, electromyogram amplitude, brain states, and eye movements curve. toREM, tonic REM. phREM, phasic REM. Sleep pattern of representative data: Wake, 4.3 min, NREM, 24.8 min, tonic REM, 4.3 min, phasic REM, 6.7 min. Bursts of eye movement were indicated by arrows. **(B)** Example of EEG, EMG, and eye movements raw traces during tonic REM **(left)** and phasic REM **(right)**. Bursts of eye movement were indicated by arrows. **(C)** PCA analysis for tonic and phasic REM. Blue dots, phasic REM. Green triangles, tonic REM. **(D)** Averaged power spectra during tonic REM and phasic REM. The red asterisk indicated statistically significant (two-way repeated-measures ANOVA followed by *post hoc* comparison with Tukey’s correction). Bin limits: 0.25 Hz. **(E)** Average power of delta (0.65–4 Hz), theta (6–10 Hz), sigma (12–14 Hz) oscillation during tonic REM and phasic REM (Norm, normalization) (*n* = 3, student’s *t*-test). Data are expressed as mean ± SEM, *p < 0.05.

Phasic REM sleep were characterized by rapid eye movements, which could be scarcely observed during tonic REM sleep (Ermis et al., 2010; Simor et al., 2018, 2019). In order to understand REM sleep, we analyzed eye movements during REM sleep, and examined the distribution of eye movement during all REM sleep, we found that about 43.2% of REM epochs did not have any eye movement (Supplementary Figure 6). To distinguish tonic REM sleep from phasic REM sleep status according to the frequency of eye movements, we define tonic REM sleep as the time with no eye movement occurred in 4-s epochs, while phasic REM sleep had eye movements occurred in 4-s epochs (Figures 5A,B). We further performed PCA analysis on the EEG and eye-movement data from already defined phasic and tonic REM and confirmed that phasic REM and tonic REM were distinct sleep states (Figure 5C). We further analyzed the oscillation power of different frequencies, and found that compared to tonic REM, phasic REM exhibited significant higher oscillation power at 0.50 Hz and lower oscillation power at 1.50–7.25 Hz and 9.50–12.00 Hz (Figure 5D). But we did not find significant difference in delta (0.65–4 Hz), theta (6–10 Hz), or sigma band (12–14 Hz) between phasic REM and tonic REM sleep (Figure 5E). These data suggested that tonic REM sleep and phasic REM sleep had different features in eye movements, as well as EEG power spectra.

## Discussion

Compared to other methods for measuring eye movements, the magnetic eye tracking provides reliable and accurate measurement of eye movements during sleep–wake cycle in mice. In this study, we developed a device for conducting eye movements and simultaneous EEG/EMG recording during sleep–wake cycle in mice. This device could track eye movements with high temporal-spatial resolution. Using this device, we discriminated phasic REM and tonic REM though eye movements, EEG and EMG data in mice and found different EEG power spectra between phasic REM and tonic REM.

As we know, the frequency of eye movements during REM sleep was higher than that during NREM sleep. Previous studies reported that during wake, REM and NREM sleep, the average frequency of eye movements in mice was about 0.90, 0.25, and 0.05 Hz, respectively, which was recorded by EOG (Fulda et al., 2011; Gutierrez Herrera et al., 2019). And we found that the average frequency of eye movements in three states is 1.61, 0.87, and 0.38 Hz, respectively. This might be due to high spatial and temporal resolution of magnetic sensor. In human, the frequency of eye movements was 3.7 ± 0.8 per minutes, and almost zero during NREM sleep (Andrillon et al., 2015), which is much lower than the frequency which we observed in mice. Meanwhile, magnetic eye tracking did not require head fix and is not limited by eyeball size. And we found that implant of magnet into conjunctiva did not affect retinal structure and RGC numbers. Thus, magnetic eye tracking is more suitable for measuring eye movements during sleep–wake cycle in mice.

Rapid eye movement sleep occupies about 20% of nighttime sleep in health human and is characterized as atony of skeletal muscles and bursts of eye movements, reduced amplitude and fast frequency of cortical EEG, compared to non-REM sleep (Siegel, 2005). Recent studies indicated that REM sleep was not a uniformly desynchronized state and could be divided into two microstates, phasic REM and tonic REM. The phasic REM sleep is characterized by bursts of ocular movements, while tonic REM sleep are more quiescent. Many researches focused on the functions and mechanisms of REM sleep, but few of them looked into phasic REM and tonic REM in mice (Brankačk et al., 2012; Simor et al., 2016). Our device could measure eye movements during sleep in mice, providing a powerful tool to exploring these two microstates of REM sleep.

Phasic REM and tonic REM were different neural states with different arousal thresholds, spontaneous oscillation activity and seems contribute differently to REM sleep dysfunctions in neurological and psychiatric disorders. In human, phasic REM sleep showed higher threshold for awakening than tonic REM sleep, and event related potentials elicited by external stimuli was reduced in phasic REM sleep (Brankačk et al., 2012), but partially reinstated during tonic REM sleep, suggesting tonic REM sleep may be more vulnerable for detecting potential danger cues. In spontaneous oscillation activity, tonic REM sleep showed higher activity in theta, beta, and alpha band, and lower activity in delta and gamma band compared to phasic REM (Simor et al., 2020). In our research, we found that tonic REM showed decreased oscillation power at 0.50 Hz and increased oscillation power at 1.50–7.25 Hz and 9.50–12.00 Hz than phasic REM. The amounts of tonic REM and phasic were varied even in the same person in different days (Tan et al., 2017). And we did not find temporal pattern of tonic and phasic REM yet in mice. These findings markedly suggested that phasic and tonic REM sleep are different microstates in REM sleep, and studying REM sleep microstates would provide new insights for functional implications of REM sleep.

Eye movements during sleep have not been sufficiently characterized in mice so far. Our device could differentiate the direction of eye movements during sleep, such as to nasal or to temporal. However, eye movements toward other directions could not be precisely characterized by the current version of this device. Additional magnetic sensors would be helpful in tracking eye movements in two- or three-dimensions during sleep. Meanwhile, the eye movement signals recorded by magnetic sensor was susceptible to interference from external magnetic fields, and this may reduce the signal-to-noise ratio under some circumstances. Proper magnetic field shielding is necessary for the recording of high-quality eye movement signals during sleep. Previous studies set magnet threshold as 5 mV/ms to detect movement artifacts. In our study, 9.5% of identified eye movements surpassed this threshold (Supplementary Figure 6B) and were considered as artifacts. In addition, there was no authorized definition of eye movements in previous studies, the parameter used for defining eye movements from the magnet signals should be regarded with caution. Meanwhile, the percentage of the phasic REM was higher than tonic REM in human (Arnaldi et al., 2016). We found that 43.2% of REM epoch has no eye movement (defined as tonic REM) in mice, exhibiting a similar fraction as that in human (Supplementary Figure 6). There was no peak in the distribution of number of eye movement in Supplementary Figure 6, indicating that there is no signature burst pattern that defines phasic REM. Hence, we did not use burst of eye movements to define phasic REM in mice.

Thus, our study provides a simple, cheap and easily made device to record eye movements during the sleep in mice, providing a good way to explore REM sleep microstates. By combining genetic tools in mice, this device would be helpful to unveil neural mechanisms of phasic and tonic REM sleep and provide novel insights into pathophysiology of sleep disorders.

## Materials and Methods

### Animals

Animal care and experiments were performed in accordance with the National Institutes of Health Guide for the Care and Use of Laboratory Animals and were approved by the Animal Care and Use Committee of Shanghai Medical College of Fudan University. Magnetic eye tracking was performed in Wild-type (C57BL/6J) mice, obtained from the Slac Laboratory Animal Co. (Shanghai, China). All mice were raised and bred at 22°C, 12-h light/dark cycles and room humidity was controlled at 50%. Each mouse was used for magnetic eye tracking in OKR, VOR, and sleep.

### Device Preparation

The NdFeB magnet was customized in the factory (La La Magnet, China). The design of device coupled with sensor was drawn by EDA-Software (Li Chuang EDA, China). PCB stator, PCB adaptor and FPC connector were self-designed for magnetic sensor (HMC 1512) connection. First, the silver wires which used for recording EEG and EMG signals were soldered to the PCB stator (Supplementary Figure 7A), then the magnetic sensor was soldered to the PCB stator on top of silver wires (Supplementary Figure 7B). Meanwhile, the FPC connector was solder to the PCB adaptor (Supplementary Figure 7C). Afterward, PCB adaptor was put on PCB stator perpendicularly, and the solder joints are welded respectively (Supplementary Figure 7D). After *in vitro* test, the epoxy resin adhesive (TRA-BOND F123 BIPAX, THORLABS, Inc., United States) was used to cover solder joints for waterproofing (Supplementary Figure 7E).

### Surgery

Surgeries were processed while mice were anesthetized by isoflurane. The NdFeB magnet of size 0.75 mm × 1.5 mm was firstly implanted into the conjunctiva in a single eye of mice. In the week after implantation, eye drops (Tobramycin Dexamethasone Eye Drops, Belgium) were given to mice every day to reduce inflammation and swelling after magnet implantation. The eye was slightly squeezed out of the socket, and eyelids were sutured in the middle to fully expose the eyeball for magnet implantation. Using ophthalmic scissors, the conjunctiva was dissected to form a pocket for fixing the magnet on the temporal side of the eye. Then the magnet was pressed into the pocket using Copper tweezers. And the magnet was implanted with the N–S axis aligned roughly perpendicular to the horizontal eye axis. Afterward, the magnetic sensor was fixed on the skull of mice. A head-bar was implanted on the cranial bone on top of head for immobilizing the head to recording eye’s position. Then the magnetic sensor weld with connector were adhered to the skull with Metabond (Sun Medical Company, Ltd., Japan). The magnetic sensor was fixed on the skull directly above the implanted magnet, the surface of which roughly parallel to the plane of nasal-temporal eye and to the plane of magnet N–S axis. The sensor was secured as close as possible to the magnet to obtain enough signal. Magnetic signals were amplified by Amplifier (Brownlee Precision Model 410, United States), and the maximum output of the amplifier is 5 V. We amplified the magnetic signals for 10× *in vitro* experiments, 60× in VOR, OKR, and spontaneous eye movement recording, and 90× in recording eye movements during sleep. Data from one mouse with magnetic signal saturated were excluded in all the experiments.

To differentiate the states during sleep–wake cycle, mice were implanted with EEG and EMG electrodes. Two stainless steel screws were inserted on the top of left and right skull at anteroposterior (AP) ++1.5 mm, mediolateral (ML) +1.5 mm and AP -3.5 mm, ML 3 mm, respectively. Two EMG electrodes were inserted into the neck musculature.

### Polysomnographic Recording

Eye movements recording during sleep–wake cycles was carried out in homemade sleeping box for 24 h one week after surgery. EEG and EMG electrodes connected to an amplifier (A-M systems), together with magnetic sensor channel, were digitized and stored by Spike2 (Power 1401, CED, United Kingdom). The magnetic sensor was supplied with a voltage of 3.3 V through an Arduino Uno microcontroller (Arduino, Italy). Magnetic signals were sampled at 9600 Baud (about 120 Hz), and the EEG and EMG signals were sampled at 128 Hz. Eye movements were detected through the magnetic signals, which were smoothed by averaging every 11 data points [taken at 9600 Baud (about 120 Hz)]. We set the detection threshold for eye movement as mean ± 2SD based on the smoothed data, and the duration for each eye movement should be less than 1.5 s. When detecting eye movements during awake, the mean and SD was calculated from the total recording during awake. While detecting eye movements during REM and NREM, the mean and SD was calculated from total recording during sleep, including REM and NREM.

### Immunohistochemistry

The eyeball was squeezed out from eye socket and retina was separated from iris with tweezers and Ophthalmic scissors. Then retina was fixed in 4% PFA for 6–9 h at 4°C. Ten percent (till sinking bottom, room temperature), 20% (till sinking bottom, room temperature, and 30% (overnight, 4°C) sucrose were sequentially used to dehydrate the retina. The retina was embedded in Optimal Cutting Temperature Compound (OCT) compound (Sakura) and stored at −80°C for more than 1 h before transferred to Frozen slicer machine (Leica CM 1950, Leica, Germany). Retina was cut at fourteen micrometers onto microslide and the slices were washed three times for 15 min in 0.05 M TBS to wash away OCT. After immersed in 0.5% Triton X-100 for 30 min, the slices were blocked in 10% Donkey serum for 2.5 h at room temperature and incubated in primary antibodies for 16–18 h at 4°C [Brn3a Goat, Santa Cruz Biotechnology, Inc., United States (sc-31984), 1:300], caspase [Rabbit, Cell Signaling Technology, United States (#9661),1:400)]. The slices were washed 3–5 times (each for 10 min) in TBS and secondary antibodies (Jackson ImmunoResearch Laboratories, Inc., United States) incubated at room temperature for 2.5 h in the dark. After removing secondary antibodies, the slices were stained in 1:3,000 DAPI solution for 6–10 min and washed three times (each for 15 min) in TBS. Finally, the slices were air dried and mounted. And fluorescent images were taken by an epifluorescence microscope (Olympus, Japan).

### Histologic Examination

For HE staining, the slices were washed for 2 min. Then the slices were stained in Harris hematoxylin solution for 3 min and washed in running tap water for 30 s. The slices were stained in Eosin solution for 20 s and differentiated in 1% acid alcohol for 30 s. Slices were blued in saturated lithium carbonate solution for 30 s and washed in running tap water for 30 s. The slices were dehydrated through 70% alcohol, 2 changes of 95% alcohol and absolute alcohol for 30 s each and cleared in xylene for 30 s. Finally, the slices were air dried and mounted with neutral resin.

### Optokinetic Stimulation

The horizontal OKR was evoked by moving horizontal grating in a virtual reality system (PhenoSys, Germany). The mouse was restrained on a ball with five screens around the mouse, which could cover the mouse’s visual field. Screens could present a vertical grating which drifted clockwise or counterclockwise. The mouse head was fixed at the center of the platform with the nasal and temporal corners of the eye leveled. Visual stimuli were given by PsychoPy running in python. The movement of the magnet-implanted eye was monitored through a high-speed infrared (IR) camera (JAI, Denmark) and an infrared light.

### Vestibular Ocular Stimulation

The mouse was fixed on a turntable with different velocity. The mouse head was placed above the center of the turntable with a holder. The IR camera and IR light were fixed on the turntable, relatively stationary to the eye of mouse to record the eye position.

### Eye Movement Trajectory Identification

For images of recorded eyeball, we used imread in MATLAB to read eye movement images. The pupil was identified by thresholding pixel values to determine the center in MATLAB and the motion trajectory of eyeball was acquired through the relative position of pupil. The eye position was measured by establishing a coordinate system with the nasal-temporal side as the *x*-axis. According to the data, including OKR, VOR, and spontaneous eye movement recorded by high-speed camera of four mice, we chose the magnet signal with the pupil in the center of orbit as zero in each mouse to calibrate the corresponding data during recording. We compared the values between the camera signal and the magnet signal by regression, on absolute amplitude values after calibration for once. Afterwards we recorded eye movements during sleep. We manually labeled the pupil of each frame by a single scorer, and then compared with the data identified by MATLAB procedure, and used Python for correlation analysis (Supplementary Figure 8). Finally, we have reviewed the video of mice eye movement artificially to ensure that the results of MATLAB procedure analysis are correct.

### Visual Acuity and Contrast Sensitivity Measurement

To test the visual acuity and contrast sensitivity of mice implanted with magnets, we developed a system. It was comprised of: (1) 4 LCD screens to give visual stimulation for mice, (2) a mice platform (height: 6 cm, radius: 4 cm) was completed by 3D printing and placed in the center of the space, (3) a camera recorded the experiments on top of the system. Visual stimuli were given by PsychoPy running in python. Each visual stimulus consists of two directions (0°, 180°), and the duration of each direction is 10 s, which occurs three times alternately. If the head of mice moves slowly with grating, the mice can recognize the current stimulus.

### Sleep State Analysis and Statistics

Electroencephalogram, EMG, and Fast Fourier Transform data was performed in SleepSign software (Version 2.0, KISSEI COMTEC). We extracted delta (0.65–4 Hz), theta (6–10 Hz) and sigma (12–14 Hz) EEG power by SleepSign. Brain state was assessed in 4-s epochs, and the sleep state of each epoch was automatically determined by SleepSign and manually verified. NREM sleep was identified as epochs with delta-dominant EEG, low EMG power, and low theta/delta EEG power ratio. REM sleep was identified as epochs with theta-dominant EEG, low EMG power, and elevated theta/delta EEG power ratio. Wake epochs were identified as periods with elevated EMG power (Chen et al., 2016). For each 4-s epoch, the EEG power from 0 to 25 Hz was first calculated respectively, and Power_max_ and Power_min_ were obtained. Normalized power of i Hz from 0 to 25 Hz were calculated as $\frac{{\text{Power}}_{i\,\mathrm{Hz}}\,-{\mathrm{Power}}_{\text{min}}\,}{{\text{Power}}_{\text{max}}\,-{\mathrm{Power}}_{\text{min}}\,}$ (Mohammadi et al., 2015).

Statistics of sleep data was based on Numpy and Pandas in Python. Heat map of EEG, EMG and eye movement curve were drawn in Python using Seaborn and Matplotlib. The analysis of data in histograms and graphs was performed using Origin version 2017 (OriginLab, United States).

### PCA Analysis and REM Classification

The classification of Tonic REM and Phasic REM, we recorded the eye movements frequency of three mice during REM stage. At the same time, the power of delta, theta and sigma was counted by SleepSign software. Based on these four types of data, we have used Python’s sklearn to do PCA analysis. And according to the classification criteria of tonic REM and phasic REM in human beings, the classification of tonic REM and phasic REM was according to the eye movements occurred during REM sleep. Phasic REM was defined if eye movements were detected at 4-s epochs, and tonic REM was defined if at 4-s epochs with no eye movements occurred.

## Data Availability Statement

The original contributions presented in the study are included in the article/Supplementary Material, further inquiries can be directed to the corresponding author/s.

## Ethics Statement

The animal study was reviewed and approved by Animal Care and Use Committee of Shanghai Medical College of Fudan University.

## Author Contributions

BY and JZ conceived the experiments and wrote the manuscript. QM and XT conducted all the experiments. XT participated in the device fabrication. CJ participated in sleep data analysis. YX participated in EEG and EMG recording during sleep. All authors contributed to the article and approved the submitted version.

## Conflict of Interest

The authors declare that the research was conducted in the absence of any commercial or financial relationships that could be construed as a potential conflict of interest.

## Supplementary Material

Supplementary Figure 1 Range of the signal by magnetic sensor in vitro. **(A)** The schematic diagram of the relative position of the magnet and sensor in vivo **(B)** The schematic showed the relative position and orientation of the magnet and magnetic sensor. **(C)** The magnetic signal when the horizontal distance between magnet and magnetic sensor was varied from 0 mm to 6 mm.

Supplementary Figure 2 The correlation between magnet signal and the video-oculography in the other three mice. **(A-C)** The correlation of Mouse 1 to 3 besides the representative mouse in Figure 2E. Left, the correlation during optokinetic reflex. Middle, the correlation during vestibular ocular reflex. Right, the correlation during spontaneous eye movement.

Supplementary Figure 3 The corneal status before and after magnet implant in the other three mice in addition to the representative mouse in Figure 3A.

Supplementary Figure 4 The magnet implant was in place three months after implant.

Supplementary Figure 5 The total daily (24 h) amounts of wake, REM and NREM in control mice (*n* = 4 mice) and the mice with magnet implant (*n* = 4). Each mouse was recorded 24 hours. Average sleep pattern of mice with magnet implant in 24 hours: Wake:18.9 h, NREM: 4.4 h, REM: 0.7 h. Average sleep pattern of control mice in 24 hours: Wake: 20.7 h, NREM: 2.8 h, REM: 0.5 h.

Supplementary Figure 6 The distribution of eye movements and speed. **(A)** The distribution of eye movements during all REM epochs (4 s). **(B)** The distribution of eye movements speed during sleep and wake phase (n = 3). Each data point is separated by 0.01 mV/ms. **(C)** The distribution of eye movements during all REM epochs (10 s).

Supplementary Figure 7 Procedure for device fabrication. **(A)** Four silver wires were soldered to the PCB stator. **(B)** The magnetic sensor was soldered to the PCB stator. **(C)** The FPC connector was solder to the PCB adaptor. **(D)** PCB adaptor was put on PCB stator perpendicularly, and the solder joints are welded respectively. **(E)** The epoxy resin adhesive was used to cover solder joints.

Supplementary Figure 8 Validation of pupil tracking by MATLAB procedure and by manual scoring. **(A)** The schematic of validating pupil position manually. **(B)** The raw trace of relative pupil position by MATLAB procedure (gray line) and manual (orange line). 700 photographs (about 14 s of OKR recording from one mouse) were selected for manual pupil labeling. **(C)** The correlation between manual scoring and MATLAB procedure in tracing pupil position.
