# Supplementary material for: Tracking Eye Movements During Sleep in Mice
Source: Front Neurosci. 2021 Feb 25;15:616760. doi: 10.3389/fnins.2021.616760 (PMC7947631; doi:10.3389/fnins.2021.616760)
Supplement: Supplementary Figure 1 — Range of the signal by magnetic sensor in vitro. (A) The schematic diagram of the relative position of the magnet and sensor in vivo (B) The schematic showed the relative position and orientation of the magnet and magnetic sensor. (C) The magnetic signal when the horizontal distance between magnet and magnetic sensor was varied from 0 mm to 6 mm. [file Data_Sheet_1.docx]

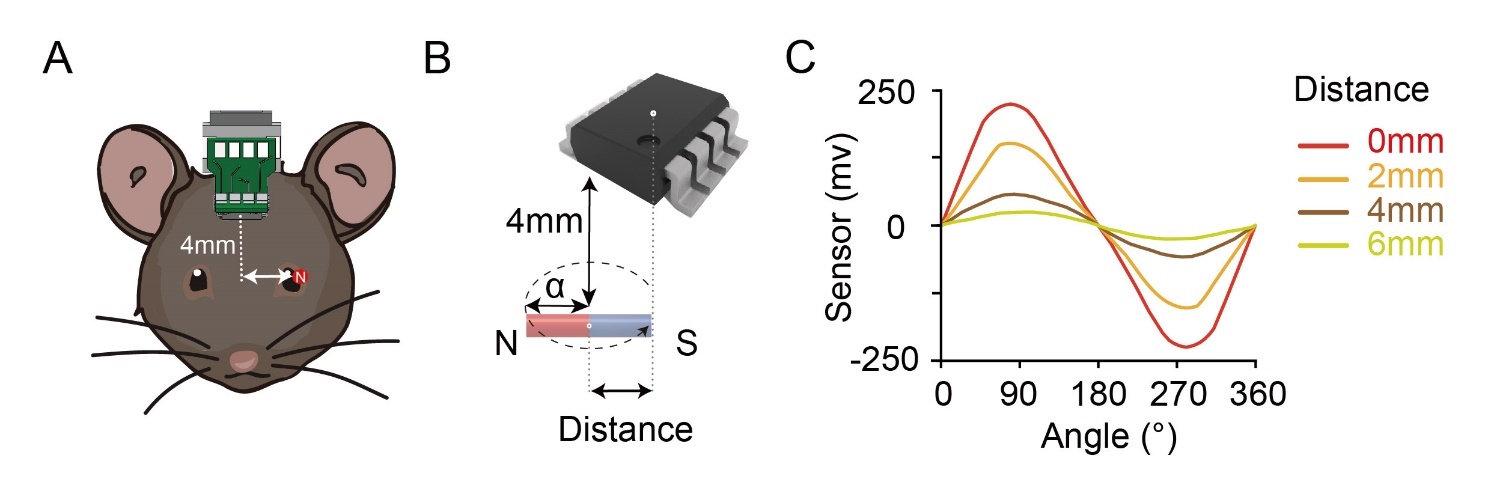


**Figure S1.** **Range of the signal by magnetic sensor in vitro.** (A) The schematic diagram of the relative position of the magnet and sensor in vivo (B) The schematic showed the relative position and orientation of the magnet and magnetic sensor. (C) The magnetic signal when the horizontal distance between magnet and magnetic sensor was varied from 0 mm to 6 mm.


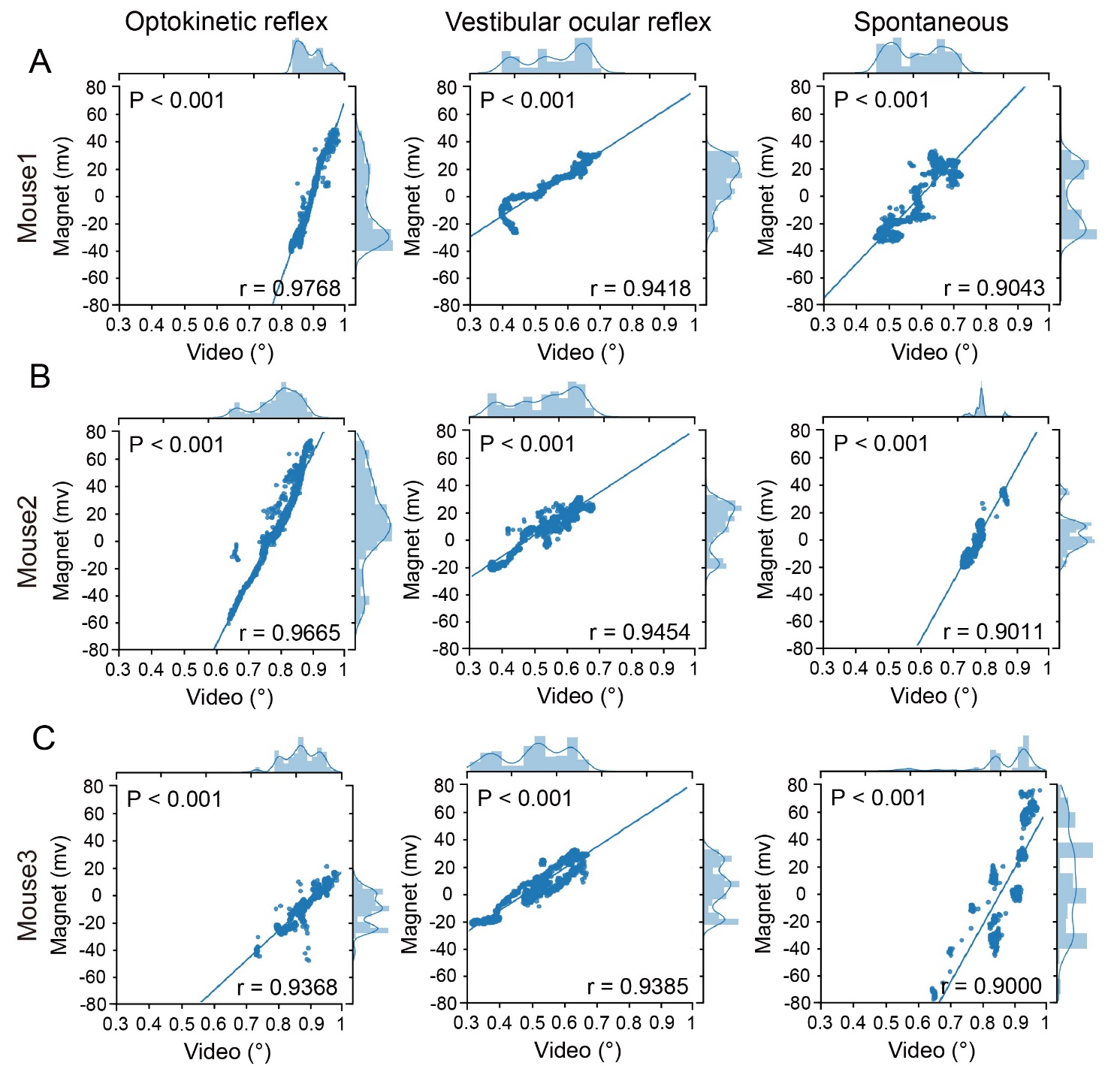


**Figure S2.** **The correlation between magnet signal and the video-oculography in the other three mice.** (A-C) The correlation of Mouse 1 to 3 besides the representative mouse in Figure 2E. Left, the correlation during optokinetic reflex. Middle, the correlation during vestibular ocular reflex. Right, the correlation during spontaneous eye movement.


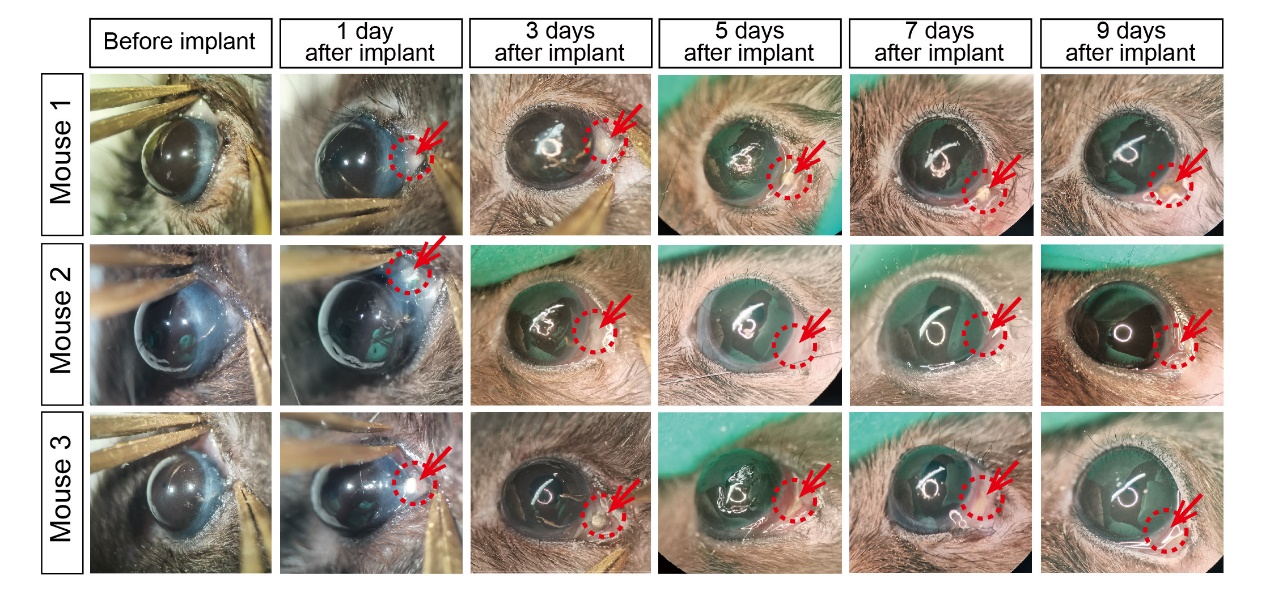


**Figure S3. The corneal status before and after magnet implant in the other three mice in addition to the representative mouse in Figure 3A.**


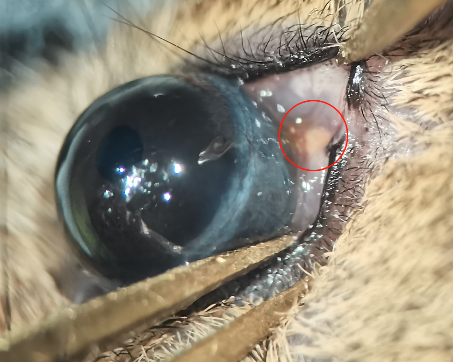


**Figure S4. The magnet implant was in place three months after implant.**

**
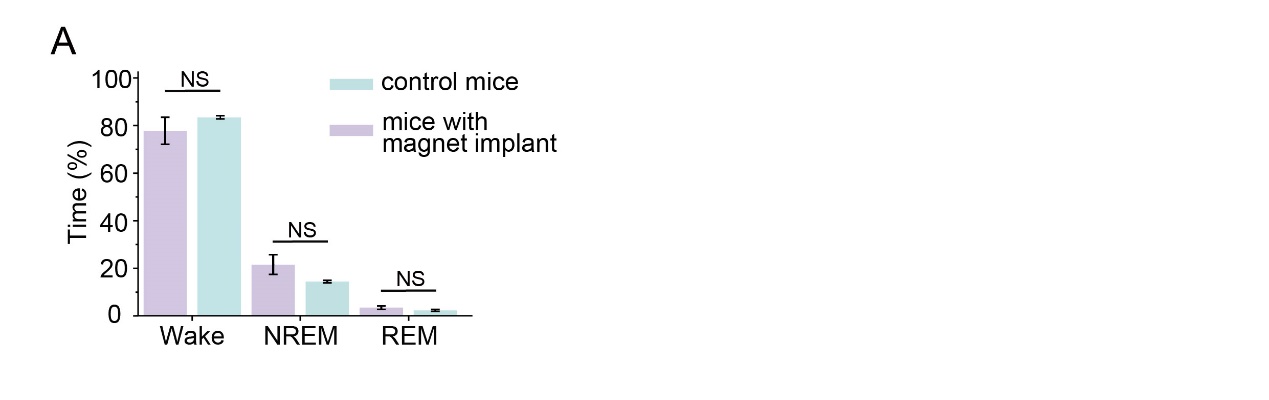
**

**Figure S5.** **The total daily (24 hours) amounts of wake, REM and NREM in control mice (n = 4 mice) and the mice with magnet implant (n = 4).** Each mouse was recorded 24 hours. Average sleep pattern of mice with magnet implant in 24 hours: Wake:18.9 h, NREM: 4.4 h, REM: 0.7 h. Average sleep pattern of control mice in 24 hours: Wake: 20.7 h, NREM: 2.8 h, REM: 0.5 h.


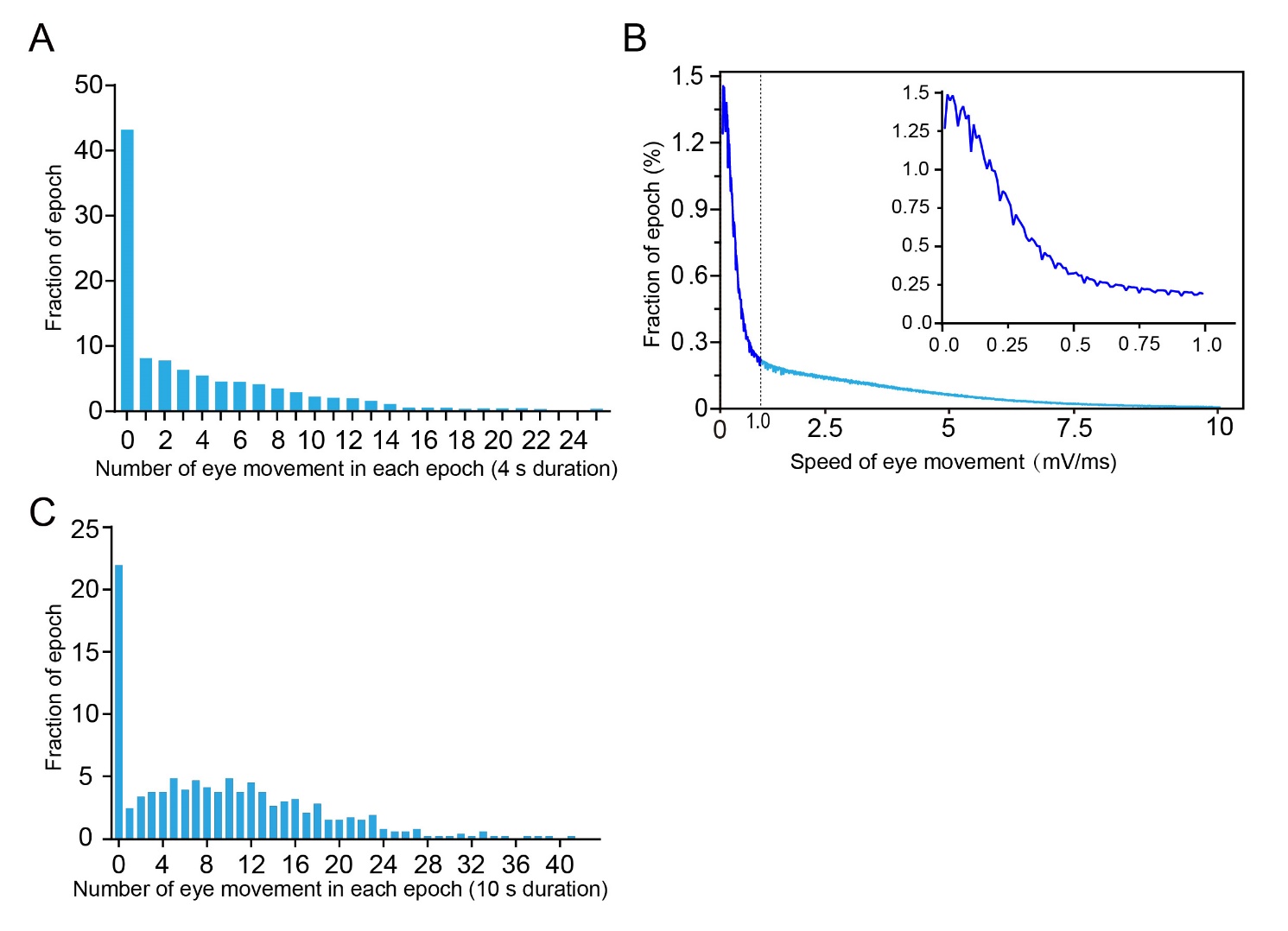


**Figure S6.** **The distribution of eye movements and speed.** (A) The distribution of eye movements during all REM epochs (4 s). (B) The distribution of eye movements speed during sleep and wake phase (n = 3). Each data point is separated by 0.01 mV/ms. (C) The distribution of eye movements during all REM epochs (10 s).

**
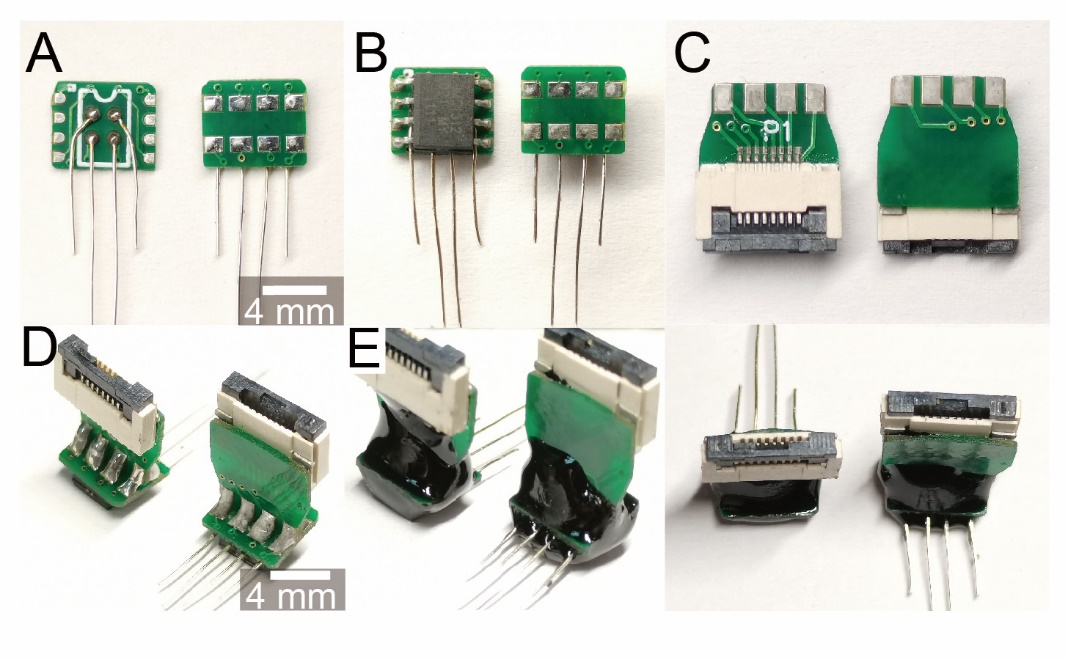
**

**Figure S7. Procedure for device fabrication.** (A) Four silver wires were soldered to the PCB stator. (B) The magnetic sensor was soldered to the PCB stator. (C) The FPC connector was solder to the PCB adaptor. (D) PCB adaptor was put on PCB stator perpendicularly, and the solder joints are welded respectively. (E) The epoxy resin adhesive was used to cover solder joints.

**
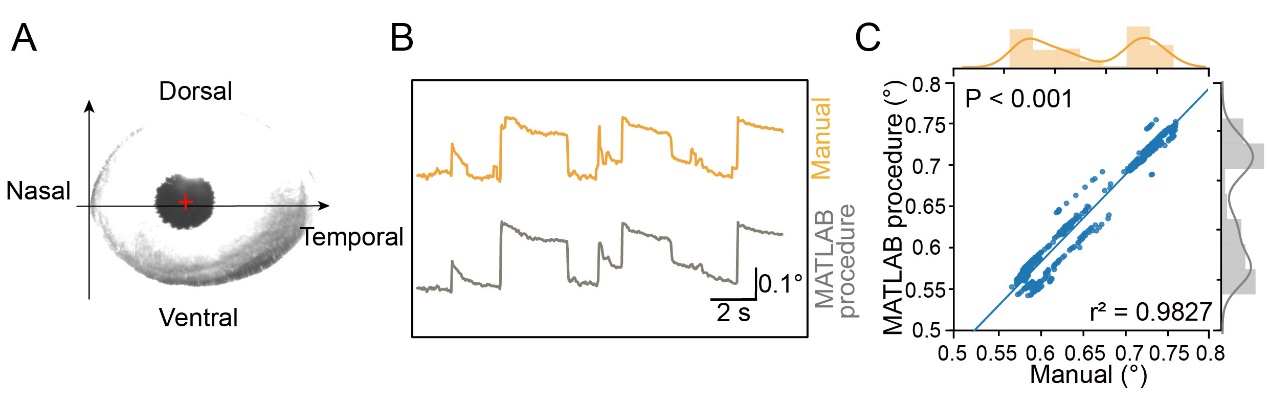
**

**Figure S8. Validation of pupil tracking by MATLAB procedure and by manual scoring.** (A) The schematic of validating pupil position manually. (B) The raw trace of relative pupil position by MATLAB procedure (gray line) and manual (orange line). 700 photographs (about 14s of OKR recording from one mouse) were selected for manual pupil labeling. (C) The correlation between manual scoring and MATLAB procedure in tracing pupil position.
